# Demographic profile and clinical picture of patients presented with Paraphenylene Diamine (PPD)/ Kala Pathar poisoning at a District Teaching Hospital

**DOI:** 10.12669/pjms.37.5.4251

**Published:** 2021

**Authors:** Sheraz Akbar, Zahid Kamal Siddiqui, Rana Aamir Diwan, Muhammad Hassam Rehm

**Affiliations:** 1 Dr. Sheraz Akbar (MBBS), Demonstrator Community Medicine Department, Sahiwal Medical College (SLMC), Sahiwal, Punjab-Pakistan; 2 Dr. Zahid Kamal Siddiqui (MBBS, FRCS, FCPS), Professor of Ophthalmology and Principal, Sahiwal Medical College (SLMC), Sahiwal, Punjab-Pakistan; 3 Dr. Rana Aamir Diwan (MBBS, MPH), Professor and Head of Community Medicine Department, Sahiwal Medical College (SLMC), Sahiwal, Punjab-Pakistan; 4 Dr. Muhammad Hassam Rehm (MBBS, MHM), Assistant Professor Community Medicine Department, Sahiwal Medical College (SLMC), Sahiwal, Punjab-Pakistan

**Keywords:** Suicide, deliberate self-harm, Paraphenylene Diamine (PPD), kala Pathar, Cervico-facial edema, Tracheostomy

## Abstract

**Objectives:**

To document demographic profile, clinical features and management of patients presented with PPD/Kala Pathar poisoning at District Teaching Hospital Sahiwal, Pakistan.

**Methods:**

This cross-sectional study utilized data for cases of PPD poisoning presented at study place from 1^st^ July 2019 to 30^th^ June 2020. Relevant information was recorded on a proforma.

**Results:**

A total of 111 cases were included in study. Mean age was 23.01 ± 7.24 years. Majority of cases were observed in females (82%) and majority presented from rural areas (87.4%). Cervico-facial edema (78.4%) and respiratory distress (66.7%) were the most common findings. Evidence of some level of organ damage was recorded in following manner: kidneys (44.1%), musculoskeletal (50.5%) and cardiac (45.9%). Tracheostomy was carried out in 47.7% cases and dialysis in 11.7% cases. All cases ingested PPD in raw form. Mortality rate was 50.5%.

**Conclusion:**

PPD poisoning is associated with high rate of morbidity and mortality. Effective clinical management requires multidisciplinary approach. Measures to restrict access to this means of suicide are urgently needed. We need to set up a surveillance system for cases of attempted suicide.

## INTRODUCTION

Suicide is the second leading cause of death, after road traffic injuries, among young people aged 15-29 years.^1^ An increasing trend in ingestion of PPD as a means of intentional self-harm has been reported in some parts of Africa and Asia including Pakistan.^2^-^5^ PPD is a chemical substance used in various industries like cosmetics. Raw concentrated form of this chemical is also mixed with henna to blacken/darken its shade. PPD ingestion in this raw powdered form has seen a surge in rural communities.^4^-^6^

PPD can cause contact dermatitis in susceptible individuals.^7^ Major systemic toxicity occurs with ingestion and its severity is dose-dependent.^7^,^8^ Clinical manifestations include angioedema (of face and throat mainly) leading to respiratory distress and dysphagia. Rhabdomyolysis and intravascular hemolysis are also seen and these may lead to acute renal failure. Hepatic necrosis, myocarditis and convulsions are some other manifestations. Mortality is high with PPD ingestion. This initially results from asphyxia and later in clinical course from renal failure. Ventricular arrhythmias also constitute an important mode of death in these patients. Management is largely supportive as there is no antidote. Mainstay of treatment is urgent tracheostomy, forced alkaline diuresis, steroids and antihistamines.^4^-^6^,^9^ Early gastric lavage has also been found to reduce mortality.^10^

Reported rapid rise in PPD poisoning cases at study place prompted the conception of this study. It attempts to document socio-demographic profile of cases presented at study place and clinical picture of PPD poisoning. This study will help us identify high risk groups, subsequently leading to targeted preventive interventions at community level. This will also lead to better patient management by facilitating health professionals to identify potential gaps in care in local settings and in due course rectify and refine patient management protocols.

## METHODS

This cross-sectional study was conducted at District Teaching Hospital Sahiwal, after approval of ethical review board of this institution (Ref: 53A/DME/SLMC/SWL, Dated: 19-09-2020). Study incorporated data for all patients of PPD poisoning presented at study place from 1^st^ July 2019 to 30^th^ June 2020. Record of patients was accessed through proper channel and relevant data were retrieved.

All patients presented with history suggestive of PPD poisoning and/or clinical findings of cervico-facial edema with no alternate diagnosis were labelled as cases of PPD poisoning. Information on following variables could be retrieved from existing data: gender, age, marital status, residential area, form of PPD ingested (raw versus hair dye), presenting and subsequent clinical features, whether management included tracheostomy and dialysis, duration of hospital stay and clinical outcome (death, recovery, leave against medical advice). Assessment of organ system damage to some level was based on clinical features and laboratory findings.

Statistical package for social sciences (SPSS) version 25 was used for data presentation and analysis. Frequencies and percentages were calculated for categorical variables and mean and standard deviation for continuous variables. Association of clinical features with outcome was assessed using Chi-square and Fisher’s exact test. A p-value less than or equal to 0.05 was considered significant.

## RESULTS

A total of 118 patients of PPD poisoning presented at health facility during specified period but record was available for 111 patients. So these 111 patients were included in study. Majority of cases (76%) presented during second half of study period i.e. from January 1^st^ 2020 to June 30^th^ 2020. All patients had history of PPD ingestion in raw powdered form, either as such or dissolved in water.

Mean age was 23.01 ± 7.24 years. Most cases were observed in females (82%), likewise majority presented from rural areas (87.4%). Frequency of cases did not differ much by marital status (52% married, 48% un-married). These findings are presented in Table-I. Using area of residence (rural) as a proxy indicator; we can allude that most cases belonged to lower socio-economic classes with less sound educational background. All cases were perceived, by attending health personnel, as cases of attempted self-harm.

**Table-I:** Demographic features of PPD poisoning cases.

Demographic feature		Frequency
Age (n=111)	≤20	53 (47.7%)
	21-30	46 (41.4%)
	≥31	12 (10.8%)
Gender (n=111)	Females	91 (82%)
	Males	20 (18%)
Area of Residence (n=95)	Rural	83 (87.4%)
	Urban	12 (12.6%)
Marital Status (n=94)	Married	49 (52.1%)
	Un-married	45 (47.9%)

Major clinical features were cervico-facial edema (78.4%) and respiratory distress (66.7%). Evidence of organ damage to some extent was recorded in following manner: musculoskeletal (50.5%), cardiac (45.9%) and renal (44.1%). Tracheostomy was carried out in 47.7% cases and dialysis in 11.7% cases. Mortality rate was 45% for all cases presented. However, 12 cases left health facility against medical advice and on excluding these cases from calculation, mortality rate was 50.5%. These findings are presented in Table-II. Average duration of hospital stay was 4.5±4.6 days and median duration was two days.

**Table-II:** Clinical features, management and outcome.

Clinical feature(n=111)	Frequency
Cervico-facial edema	87 (78.4%)
Respiratory distress	74 (66.7%)
***Organ damage to some degree (n=111)***	
Musculoskeletal	56 (50.5%)
Cardiac	51 (45.9%)
Renal	49 (44.1%)
***Management (n=111)***	
Tracheostomy	53 (47.7%)
Dialysis	13 (11.7%)
***Outcome (n=99)***	
Death	50 (50.5%)
Recovered	49 (49.5%)

Development of cervico-facial edema and respiratory difficulty showed statistically significant association with mortality (p<0.001). Contingency tables for organ damage and outcome yielded statistically insignificant results. These findings are presented in Table-III.

**Table-III:** Association of clinical features with outcome (n=99).

Variable	Outcome		Statistical association (p-value)

	Death	Recovery	
Yes	50	32	<0.001
***Cervico-facial edema***			
No	00	17	
Yes	48	24	<0.001
***Respiratory difficulty***			
No	02	25	
Yes	26	18	0.126
***Renal damage***			
No	24	31	
Yes	20	27	0.132
***Cardiac damage***			
No	30	22	
Yes	23	30	0.129
***Muscle damage***			
No	27	19	

## DISCUSSION

The most common methods for committing suicide globally are hanging, pesticides intake and use of firearms. Pesticide ingestion has been preferred means of deliberate self-harm in rural communities in Asia including Pakistan.^1^,^9^,^11^ Recent surge in PPD use as a means of deliberate self-harm in rural communities in some countries might be attributed to factors like ease of access, low cost and salty taste (as opposed to bitter taste of some other potential poisons.^2^,^13^ However in order to devise better preventive and curative interventions further research into methods used for suicide is required.^14^,^15^

We found that most patients of PPD poisoning were females (82%), likewise most presented from rural areas (87.4%). Frequency of cases did not differ much by marital status (52% married, 48% un-married). Similar female preponderance has been observed in most relevant local studies^4^,^5^,^13^, although some studies reported slightly lower frequency than our study.^6^,^7^ Other studies which commented on area of residence made similar observations.^16^,^17^ Regarding marital status, most studies which incorporated this variable found that majority of cases were unmarried (70-75%), however a study conducted by Ansari et al. reported much higher percentage of cases to be married (94%).^16^,^18^,^19^ Using (rural) area of residence as a proxy indicator, we concluded that most cases had low economic status and less sound educational background. Although there was no direct comment on mode of poisoning in our data set but apparently all cases were perceived, by attending health personnel, as cases of attempted self-harm. These finding are in line with almost all of available literature on subject. Few studies also mentioned an accidental ingestion of PPD in children.^5^-^7^,^12^,^13^,^16^ We must add that considering characteristics of PPD, it has a potential for homicidal use as well.

Recorded mean age was 23±7.24 years. Other studies reported similar findings.^5^,^7^,^16^ Chief presenting complaints were cervico-facial edema (78.4%) and respiratory distress (66.7%) and these findings were associated with high mortality as well (Table-III). Evidence of organ-system damage to some degree was recorded in following manner: musculoskeletal (50.5%), cardiac (45.9%) and renal (44.1%). In many patients outcome (mortality, discharge, leave against medical advice) was observed soon after presentation, with little time for organ damage to occur and/or to be recorded. Other studies reported similar presenting complaints but at a higher frequency. In literature there are varying figures on organ damage.^5^,^7^,^13^,^16^ However it is difficult to make a meaningful comparison since there is very little explicit mentioning of standardized definitions and uniform protocols for measuring these findings in studies conducted.

We found that tracheostomy was carried out in 47.7% cases. A study conducted in Southern areas of Punjab and KPK and another study in India reported similar findings.^13^,^20^ However many studies have reported that higher percentage (75-100%) of cases underwent tracheostomy.^4^,^5^,^6^,^16^,^19^ This discrepancy could be the result of difference in referral pattern for different health facilities or difference in quality of care provided. Lack of uniform criterion for tracheostomy could be another possible explanation (in our settings following parameters were assessed for making a decision regarding tracheostomy: dyspnea, stridor, decreased oxygen saturation and edema of face, neck and tongue). Some researchers recommend that all patients presented with PPD poisoning should undergo tracheostomy^4^,^5^,^20^ but considering some factors associated with this procedure including expertise required, invasive nature and prolonged hospital stay (along with its drawbacks) imply that it is not a straightforward decision. This issue requires further exploration with better designed studies using more comparable and precisely recorded data. Regardless of everything else there is a strong consensus in literature that the most immediate threat in these patients is airway obstruction and securing airway should be top priority. In this context clear protocols to secure airway (whether conservatively, through endo-tracheal tube, through tracheostomy or cricothyroidotomy) should be devised and adhered to. It is an uphill task requiring collaborated effort from different specialties. We found that dialysis was carried out in 11.7% of cases. A study conducted at Multan yielded similar result.^5^

Mortality rate was 50.5%, quite a high number. A study conducted at District Teaching Hospital D. I. Khan reported similar mortality rate (47.4%).^21^ Another study conducted at District Hospitals of South Punjab and South KPK revealed even more astonishing results by presenting mortality rate of 80%.^13^ Most studies in literature reported a mortality rate somewhere between 20-40%.^4^,^5^,^6^,^7^,^16^,^17^,^19^ There could be several possible explanations for this difference including a difference in referral pattern for health facilities (resulting in some kind of selection bias), difference in time of presentation since poisoning, difference in concentration (and quantity) of PPD ingested and so forth. However legitimate concern over quality of care at health facilities with relatively higher mortality rate can only be overruled with some conclusive evidence.

Time since poisoning, at reaching health facility, was available for 66 cases only (out of 111). Of these, 09 patients presented with a delay of at least 24 hours. For remaining 57 patients time estimate ranged from 01-18 hours. These numbers reflect significant time lag between poisoning and presentation at health facility and this is quite different from some other studies and could be one reason for higher mortality rate observed in our study.^4^,^5^,^7^,^16^,^21^ Mean duration of hospital stay was 4.5±4.6 days and median was two days. Most other local studies reported a much prolonged hospitalization time.^5^,^13^,^17^ One possible explanation for this shorter average duration of hospital stay in our study could be high mortality as mortality in PPD poisoning usually occurs within short duration after poisoning.

Another thought-provoking scenario emerged as we considered high mortality, late presentation and short mean duration of hospital stay in combination; a possible explanation could be the potential impact of COVID-19 pandemic on these variables. So due to COVID-19 pandemic patients (and family members) might have been hesitant to report to hospital to seek care and this might have contributed to delayed presentation. This delayed presentation combined with possibly sub-optimal health services (as most of limited resources were diverted to tackle COVID-19) might have contributed to increased mortality. This COVID-19 associated situation could also be a factor promoting early discharge and leave against medical advice. Another observation was that most cases (74%) reported during 2^nd^ half of study period (from January 1^st^ 2020 to June 30^th^ 2020). This interval closely coincides with period of COVID-19 pandemic. So this increased frequency of attempted suicides could be influenced by the COVID-19 associated lock-down and other factors. Hence COVID-19 pandemic could have potentially impacted all these variables in following manner: increase in frequency of attempted suicides, delay in seeking care, increase in mortality, shortening of hospital stay and so forth. Similar studies conducted at different centres incorporating data for same interval could help to further explore this association.

It is well established that having a history of one or more acts of attempted suicide or deliberate self-harm is the single most important predictor for future suicide attempts.^22^,23 It was so disappointing that we could not retrieve information on history of self harm before in our cases and there was no comment on whether the psychological counseling (to patient and family) was provided by attending physicians. In addition referral to psychiatrist was recommended for survivors only in few cases. Relevant studies conducted in region also failed to document these variables. These issues need to be addressed for preventing future suicide attempts in survivors.

### Limitations of the Study:

- We utilized pre-existing data for study. Since such data were not primarily meant for research purpose, so there was deficiency in completeness and quality of data with respect to research questions.
- Criteria for labelling different clinical manifestations and for recommending interventions were not explicitly mentioned in our data set and so is the case with most available literature. This lack of uniformity makes it difficult to draw meaningful comparisons.

## CONCLUSION

PPD poisoning is emerging as an important means of deliberate self-harm. It is associated with high morbidity and mortality.

### Recommendations

- AS PPD poisoning related attempted suicide is frequently being observed in rural areas of District Sahiwal and nearby Districts of Faisalabad and Okara, there is need to convince local administration to restrict access to PPD (specially its raw powdered form).
- Suicide prevention programmes at community level should be designed and implemented with help of influential figures in community including religious leaders. Policies for productive use of social media forums should also be considered including support groups formation for suicide prevention.
- As clinical management of PPD poisoning patients requires multidisciplinary effort, there is need to enhance coordination among departments in hospital settings. Treatment protocols should be further refined and standardized.
- Data documentation should be improved.
- There is a dire need to establish and maintain surveillance system for hospital-presented suicide attempts (in line with WHO recommendations).

### Authors’ Contribution:

**SA** did acquisition, analysis and interpretation of data, initial and subsequent drafting of manuscript.

**ZKS** conceived and designed the study and edited and approved final version

**RAD** did critical revision and contributed in drawing recommendations

**MHR** was involved in subsequent drafting
